# Adhiron: a stable and versatile peptide display scaffold for molecular recognition applications

**DOI:** 10.1093/protein/gzu007

**Published:** 2014-03-25

**Authors:** Christian Tiede, Anna A. S. Tang, Sarah E. Deacon, Upasana Mandal, Joanne E. Nettleship, Robin L. Owen, Suja E. George, David J. Harrison, Raymond J. Owens, Darren C. Tomlinson, Michael J. McPherson

**Affiliations:** 1 Biomedical Health Research Centre, BioScreening Technology Group, University of Leeds, Leeds LS2 9JT, UK; 2 School of Molecular and Cellular Biology, Universityof Leeds, Leeds LS2 9JT, UK; 3 Astbury Centre for Structural Molecular Biology, Faculty of Biological Sciences, University of Leeds, Leeds LS2 9JT, UK; 4 The Oxford Protein Production Facility UK, Research Complex at Harwell, Rutherford Appleton Laboratory, Harwell Science and Innovation Campus, Didcot, Oxfordshire OX11 0FA, UK; 5 Division of Structural Biology, University of Oxford, Wellcome Trust Centre for Human Genetics, Roosevelt Drive, Oxford OX3 7BN, UK; 6 Diamond Light Source, Harwell Science and Innovation Campus, Didcot, Oxfordshire OX11 0DE, UK

**Keywords:** consensus protein, high specificity binding, non-antibody-binding protein, protein–protein interaction, SUMO

## Abstract

We have designed a novel non-antibody scaffold protein, termed Adhiron, based on a phytocystatin consensus sequence. The Adhiron scaffold shows high thermal stability (*T*_m_ ca. 101°C), and is expressed well in *Escherichia coli*. We have determined the X-ray crystal structure of the Adhiron scaffold to 1.75 Å resolution revealing a compact cystatin-like fold. We have constructed a phage-display library in this scaffold by insertion of two variable peptide regions. The library is of high quality and complexity comprising 1.3 × 10^10^ clones. To demonstrate library efficacy, we screened against the yeast Small Ubiquitin-like Modifier (SUMO). In selected clones, variable region 1 often contained sequences homologous to the known SUMO interactive motif (V/I-X-V/I-V/I). Four Adhirons were further characterised and displayed low nanomolar affinities and high specificity for yeast SUMO with essentially no cross-reactivity to human SUMO protein isoforms. We have identified binders against >100 target molecules to date including as examples, a fibroblast growth factor (FGF1), platelet endothelial cell adhesion molecule (PECAM-1; CD31), the SH2 domain Grb2 and a 12-aa peptide. Adhirons are highly stable and well expressed allowing highly specific binding reagents to be selected for use in molecular recognition applications.

## Introduction

Antibodies are the most commonly used binding proteins with >240 candidates in clinical development (Reichert, 2010) and remain extremely important in scientific research, diagnostics and therapy. Nevertheless, antibodies have a number of limitations; they are large multimeric proteins that require disulphide bonds and glycosylation for stability. They are usually difficult to express in bacterial systems and are often highly sensitive to elevated temperatures. Antibody production is generally expensive, relies upon the use of animals and mammalian cell culture, while for clinical applications they must be engineered as humanised versions. Protein engineering has been exploited to develop alternative non-antibody binding proteins which mimic the molecular recognition properties of antibodies but with some improved properties.

Some 50 novel non-antibody protein scaffolds have been designed to constrain and present variable peptide sequences for protein recognition (Skerra, 2007; Gebauer and Skerra, 2009). These include designed ankyrin repeat proteins (DARPins) (Binz *et al.*, 2003), Repebodies (Lee *et al.*, 2012), Anticalins (Schlehuber and Skerra, 2005), Fibronectins (Koide *et al.*, 1998), Affibodies (Nord *et al.*, 1995) and engineered Kunitz domains (Nixon and Wood, 2006). Artificial binding proteins are in general small (<200 aa), monomeric, stable and easy to express in *Escherichia coli*. Most do not contain cysteines enabling the introduction of a cysteine for site-specific coupling of biotin, fluorescent labels, or polyethylene glycol to enhance their utility or stability. These characteristics make artificial binding proteins powerful tools capable of replacing antibodies in a range of applications including research (Wojcik *et al.*, 2010), diagnostics (Theurillat *et al.*, 2010), *in vivo* drug discovery studies (Grebien *et al.*, 2011; Parizek *et al.*, 2012) and a novel class of therapeutics (recently reviewed (Gebauer and Skerra, 2009; Wurch *et al.*, 2012)) including multivalent and/or multi-specific protein therapeutics (Carter, 2011). A potential disadvantage of small artificial proteins for therapeutic purposes is a short circulatory half-life; however, this can be overcome by fusing them to larger proteins such as albumin or by using PEGylation or PASylation (XL-Protein GmbH) or XTEN (Schellenberger *et al.*, 2009) approaches.

An important consideration when designing artificial protein scaffolds is their thermostability. In general, there is a correlation between thermal stability and other aspects of protein stability, so a highly stable scaffold enables long-term storage at ambient temperature and also broadens utility in a range of processes including heat purification and options for the storage of reagents and administration of therapeutics. Insertion of peptide loops into a scaffold often leads to a decrease in protein stability so it is desirable to select a very stable scaffold for combinatorial library generation. One approach to generate stable proteins is the consensus design concept (Steipe *et al.*, 1994). This is based on the premise that sequence conservation arises from evolutionary pressure to maintain stability elements (Mosavi *et al.*, 2002). While any natural protein is expected only to have evolved the level of stability required for it to efficiently perform its function, a consensus protein reinforces structural stability. This approach has been successfully used to improve the thermostability of enzymes (Lehmann *et al.*, 2000; Komor *et al.*, 2012), antibodies (Knappik *et al.*, 2000) as well as artificial binding proteins (Main *et al.*, 2003; Forrer *et al.*, 2004; Jacobs *et al.*, 2012).

In the present study, we describe a new artificial binding protein scaffold that we have named Adhiron. This scaffold is based on a consensus sequence of plant-derived phytocystatins, which are small (ca. 100 aa) protein inhibitors of cysteine proteases (Kondo *et al.*, 1991). This consensus protein displayed very good protease inhibitor activity and also the requirements (small, monomeric, high solubility and high stability and the lack of disulfide bonds and glycosylation sites) for a good scaffold for peptide presentation. We chose to replace inhibitory sequences within the Gln Val Val Ala Gly and Pro Trp Glu loops of the consensus phytocystatin with nine randomized amino acid positions in each loop.

To evaluate the functionality of our scaffold and the phage-display library, we chose yeast SUMO protein as a model target for screening. Four different yeast SUMO-binding Adhirons were selected and characterized for binding affinity, stability and specificity. In addition, we have selected specific binders against some 100 distinct molecules between 2011 and 2013 including, as examples, fibroblast growth factor (FGF1), cell adhesion molecule CD31, SH2 domains and a 12-aa peptide.

## Materials and methods

### Construction of the Adhiron library

**Fig. 1.**
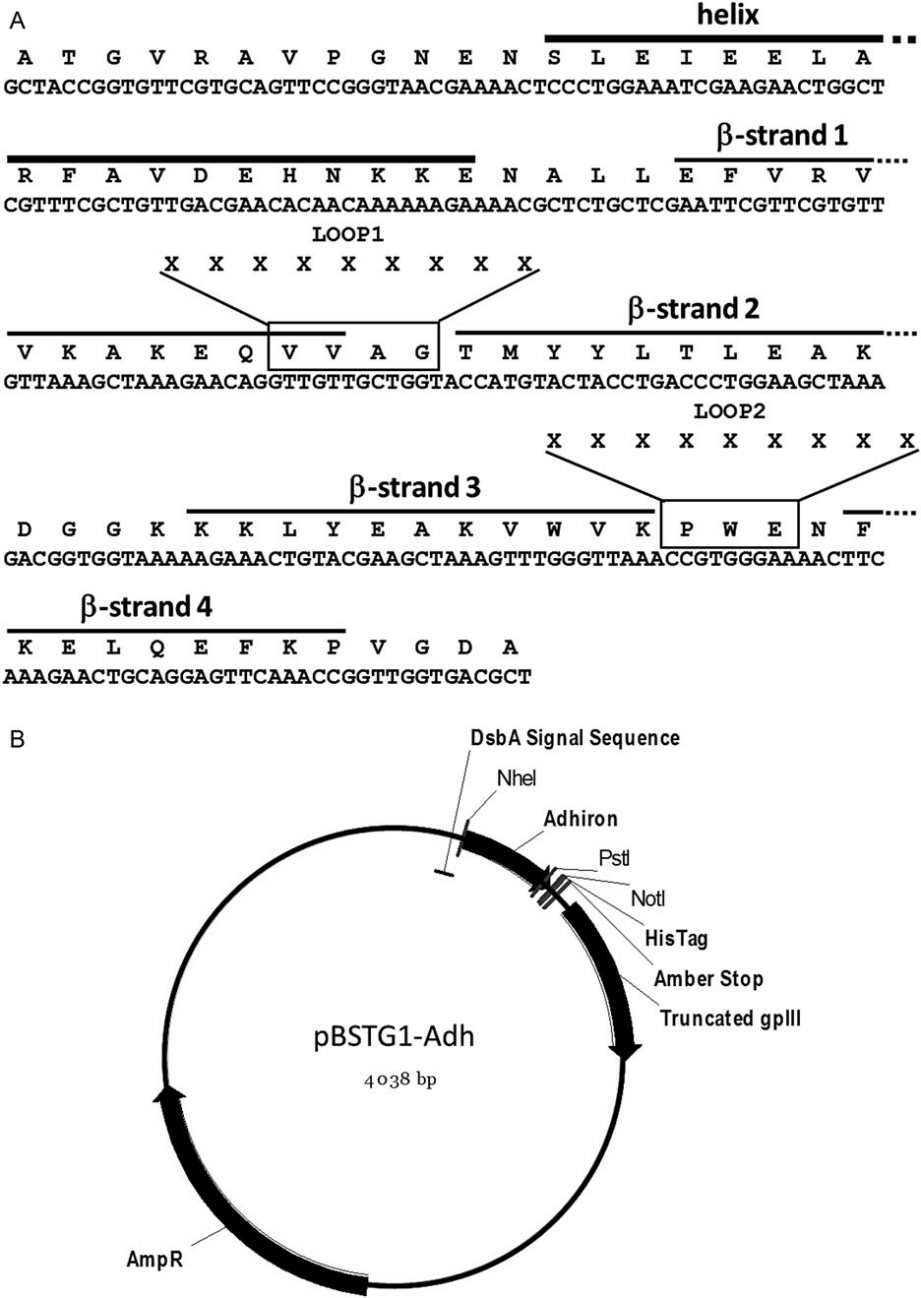
Adhiron coding region and phagemid vector. (**A**) Codon optimized coding sequence and amino acid sequence of the Adhiron92 scaffold with secondary structure elements indicated. The residues that are replaced by the nine randomized amino acids (X) to form LOOP1 and LOOP2 in the Adhiron library are boxed. In place of the N-terminal residues AlaThrGly, the original consensus sequence contained the N-terminal sequence AlaAlaLeuLeuGlyGly. (**B**) pBSTG1 phagemid vector containing the coding region for Adhiron92 indicating relevant features of the construct.

A consensus sequence derived from alignment of 57 phyocystatin sequences was developed to enhance phytocystatin properties. To design the consensus phytocystatin gene, a tBLASTN search of the Genbank database was performed using OSA-I (*Oryza sativa*; U54702), ZMA2 (*Zea mays*; D38130) and HAN1 (*Helianthus annuus*; Q10993) protein sequences as search probes. The accession codes and sources of sequences used to derive the consensus sequence are shown in Supplementary Table SI. These coding sequences were translated and aligned using the program MULTALIN (Corpet, 1988). This consensus protein was then further modified and truncated for use as a non-antibody protein scaffold, termed Adhiron, and an *E. coli* codon optimised gene was synthesised (GenScript) (Fig. 1A). The Adhiron scaffold coding region was cloned between NheI and NotI restriction sites in a phagemid vector pBSTG1 (GenBank accession number KJ474865) developed from pHEN1 (Hoogenboom *et al.*, 1991). Cloning into pBSTG1 creates a fusion coding sequence encoding a DsbA secretion signal peptide, Adhiron, TAG codon and C-terminal half of gene III of bacteriophage M13 (Fig. 1B). The resulting phagemid is referred to as pBSTG1-Adh. The in-frame amber (TAG) stop codon allows translational read-through to create an Adhiron-truncated-pIII fusion protein in a suppressor *E. coli* strains such as ER2738 (F′*proA^+^B^+^ lacIq* Δ(*lacZ*)*M15 zzf*::Tn10(TetR)/*fhuA2 glnV* Δ(*lac-proAB*) *thi-1* Δ(*hsdS-mcrB*)5) but not in a non-suppressor strains such as JM83 (*ara* Δ*(lac-proAB) rpsL* (Str^r^)[*φ80* d*lac*Δ*(lacZ)M15*] *thi*).

The Adhiron library was constructed by splice overlap extension (SOE) of two PCR products (Horton *et al.*, 1990) and all primers were synthesised by Ella Biotech. The first PCR product extended from the DsbA coding sequence to the first variable loop and was generated by the primers: Forward primer 5′-TCTGGCGTTTTCTGCGTC-3′ and Reverse primer 5′-CTGTTCTTTCGCTTTAACAAC-3′. The second PCR product introduced two nine amino acid variable regions (VRs) into the scaffold using the following primers. The PstI site used for cloning is underscored: Forward_VR 5′-GTTGTTAAAGCGAAAGAACAGNNNNNNNNNNNNNNNNNNNNNNNNNNNACCATGTACTACCTGACCCTG-3′ and Reverse_VR 5′-CTGCGGAACTCCTGCAGTTCTTTGAAGTTNNNNNNNNNNNNNNNNNNNNNNNNNNNCTTAACCCAAACTTTCGCTTCG-3′.

The degenerate positions (NNN) were introduced as trimers representing a single codon for each of the 19 amino acids excluding cysteine and there were no termination codons. PCR used Phusion High Fidelity Polymerase (NEB) at 98°C for 5 min then 20 cycles of 98°C, 10 s; 56°C, 15 s; 72°C, 15 s followed by 72°C for 5 min. PCR products were purified by gel extraction (Qiagen), used for 10 cycles of SOEing as above, then digested with NheI and PstI, gel extracted and cloned into the similarly digested pBSTG1-Ad phagemid.

Ligated products were electroporated into *E. coli* ER2738 electrocompetent cells (Lucigen). In total, 20 ml of ER2738 cells was electroporated with 126 ng of library DNA per 50 μl of ER2738 cells. After a 1-h recovery in 2TY medium, cells were grown at 37°C and 225 rpm to an OD_600_ of 0.6 in 5 l of 2TY medium. M13KO7 helper phage (10^11^) were added with shaking at 37°C and 100 rpm for 1 h followed by overnight at 25°C with kanamycin (50 µg/ml). Phage were precipitated with 6% polyethylene glycol 8000 and 0.5 M NaCl, and were suspended in 50% glycerol for storage.

### Target preparation and phage display

Yeast SUMO (ySUMO) protein was expressed in BL21 (DE3) cells using isopropyl β-d-1-thiogalactopyranoside (IPTG) induction and purified by Ni-NTA resin (Qiagen) affinity chromatography according to the manufacturer's instructions. Purity was confirmed by sodium dodecyl sulphate polyacrylamide gel electrophoresis (SDS–PAGE). The following protocols are described for yeast SUMO with an identical protocol used for the screening of other targets. Yeast SUMO was biotinylated using EZ-link NHS-SS-biotin (Pierce), according to the manufacturer's instructions. Biotinylation was confirmed using streptavidin conjugated to horseradish peroxidase (HRP). Biotinylated ySUMO was bound to streptavidin-coated wells (Pierce) for 1 h, then 10^12^ cfu pre-panned phage were added for 2.5 h with shaking. Panning wells were washed 10 times and eluted with 50 mM glycine–HCl (pH 2.2) for 10 min, neutralised with 1 M Tris–HCL (pH 9.1), further eluted with triethylamine 100 mM for 6 min, and neutralised with 1 M Tris–HCl (pH 7). Eluted phage were used to infect ER2738 cells for 1 h at 37°C and 90 rpm then plated onto LB agar plates with 100 µg/ml carbenicillin and grown overnight. Colonies were scraped into 5 ml of 2TY medium, inoculated in 25 ml of 2TY medium with carbenicillin (100 µg/ml) and infected with ca. 1 × 10^9^ M13K07 helper phage. After 1 h at 90 rpm, kanamycin was added to 25 μg/ml for overnight at 25°C and 170 rpm. Phage were precipitated with 4% polyethylene glycol 8000, 0.3 M NaCl and resuspended in 1 ml of 10 mM Tris, pH 8.0, 1 mM EDTA (TE buffer). A 2 µl aliquot of phage suspension was used for the second round of selection using streptavidin magnetic beads (Invitrogen). Yeast SUMO labelled beads were washed and incubated with pre-panned phage for 1 h then washed five times using a KingFisher robotic platform (ThermoFisher) and eluted and amplified as above. The final pan used neutravidin high binding capacity plates (Pierce), as previously described for panning round one, with phage eluted using 100 µl of 100 mM dithiothreitol. Phage were recovered from wells containing target protein and control wells to determine the level of amplification in target wells.

### Phage ELISA

Individual ER2738 colonies were grown in 100 µl of 2TY with 100 µg/ml of carbenicillin in a 96-deep well plate at 37°C (900 rpm) for 6 h. A 25 µl aliquot of the culture was added to 200 µl of 2TY containing carbenicillin and grown at 37°C (900 rpm) for 1 h. Helper phage (10 µl of 10^11^/ml) were added, followed by kanamycin to 25 μg/ml overnight and incubated at 25°C (450 rpm). Streptavidin-coated plates (Pierce) were blocked with 2 × casein blocking buffer (Sigma) overnight at 37°C. The plates were incubated with biotinylated yeast SUMO or biotinylated linker for 1 h, and 45 µl of growth medium containing the phage was added and incubated for 1 h. Following washing, phage were detected by a 1 : 1000 dilution of HRP-conjugated anti-phage antibody (Seramun) for 1 h, visualised with 3,3′,5,5′-tetramethylbenzidine (TMB) (Seramun) and measured at 610 nm.

### Adhiron protein production

The DNA coding sequences of Adhirons were amplified by PCR, restriction digested with NheI and PstI and cloned into pET11a containing the Adhiron scaffold similarly digested. Plasmid DNA was purified (Qiagen) from transformant colonies and sequenced to confirm the correct insert. Following transformation into BL21 (DE3) cells (*F^−^ ompT gal dcm lon hsdS_B_(r_B_^−^ m_B_^−^) λ(DE3* [*lacI lacUV5-T7 gene 1 ind1 sam7 nin5*]) 400 ml cultures in LB medium were induced with 0.1 mM IPTG. Cells were grown for 6 h, harvested and lysed with Bugbuster (Novagen). The clear supernatant was mixed with 500 µl of Ni-NTA resin slurry (Qiagen) for 1 h, washed three times in (50 mM PBS, 500 mM NaCl, 20 mM imidazole, pH 7.4) and eluted in 3 × 1 ml of elution buffer (50 mM PBS, 500 mM NaCl, 300 mM imidazole, pH 7.4). One hundred micrograms of the SUMO-binding Adhirons were biotinylated using NHS SS-biotin (Pierce) according to the manufacturer's instructions for use in enzyme-linked immunosorbent assay (ELISAs) and western blotting.

### ELISA analysis with purified Adhirons

Unless otherwise indicated 5 ng of target protein in PBS was absorbed onto Immuno 96 Microwell™ Nunc MaxiSorp™ plate wells overnight at 4°C. Two hundred microlitre of 3× blocking buffer was added at 37°C for 4 h with no shaking. Biotinylated yeast SUMO-binding Adhirons (Ad-ySUMO) at 0.1 µg/ml in Phosphate-buffered saline with Tween 20 (PBST) containing 2× blocking buffer were incubated in wells for 1 h with shaking. Wells were washed three times in PBST, and streptavidin conjugated to HRP (Invitrogen) diluted 1 : 1000 in 50 µl PBST was added for 1 h. After washing Adhiron binding was visualised with 50 µl TMB (Seramun) and the absorbance measured at 610 nm.

### Western blot analysis

Target protein alone or mixed with HEK293 cell lysate (20 µg) was mixed with loading buffer (60 mM Tris–Cl pH 6.8, 2% SDS, 10% glycerol, 5% β-mercaptoethanol, 0.01% bromophenol blue), boiled for 3 min, centrifuged for 1 min at 15 000*g* and then resolved in a 15% SDS–polyacrylamide gel. Proteins were transferred to PVDF membranes for 45 min at 4 W (Amersham Biosciences) and incubated for 1 h in blocking buffer (5% BSA in PBS 0.1% Tween) followed by incubation for 1 h with Ad-ySUMO (100 µg/ml diluted 1 : 1000 PBST). Bound Ad-ySUMOs were detected using streptavidin-conjugated HRP and chemiluminescence (ECL Plus kit, Amersham).

### Mass spectrometry analysis of Adhirons

Adhiron samples in PBS were buffer exchanged into 50 mM ammonium acetate using Zeba spin columns (7K MWCO; Thermo Scientific) and 20 ml samples of 20 mM protein were analysed on a Synapt HDMS (Waters UK Ltd) electrospray mass spectrometer.

### Protein–protein interaction affinity measurement

The BLitz™ (ForteBio) dip and read streptavidin biosensors were used to estimate the affinity of ySUMO binding to immobilised biotinylated Ad-ySUMO binders, according to the manufacturer's instructions. At least four readings at different ySUMO concentrations (0.25–1 mM) were used for each Ad-ySUMO and a global fit was used to calculate binding affinities. The values obtained were comparable with affinities measured using a Biacore SPR3000 instrument.

### DSC

Adhiron thermal stability was measured by differential scanning calorimetry (DSC) using a VP-DSC MicroCalorimeter (GE Healthcare). Each sample was dialysed extensively against PBS, pH 7.4 and diluted to a concentration of 1 mg/ml before heating from 10 to 100°C for Adhiron library clones or to 120°C for the Adhiron scaffold 92 and 81 amino acid proteins at an upscan rate of 90°C per hour. A buffer only scan was measured to calculate a baseline for integration. Data were fit to a one-state unfolding model and reversibility of thermal denaturation was determined by repeating the scan for the Adhiron scaffold sample without removing it from the cell.

### ITC

All experiments were carried out using the iTC200 system (Microcal) at 25°C. Typically, 0.1 mM Adhiron protein was titrated with 0.01 mM of a target protein (yeast Sumo, human Sumo1, human Sumo2) by 2.5 min injection duration to allow return of the titration peak to the baseline. After fitting the integrated exothermal peaks, *K*_D_ values were determined using the Origin program (OriginLab).

### CD studies

All circular dichroism (CD) measurements were performed on a Chirascan CD spectrometer (Applied Photophysics) at room temperature. The spectra were recorded over a wavelength range of 260–180 nm using a cuvette of 1 mm pathlength at a scan speed of 60 nm/min. The concentration of each sample was ca. 0.2 mg/ml in PBS buffer (pH 7.4). Data were further processed for noise reduction, baseline subtraction and signal averaging.

### Structure determination

Crystallization trials were set up using the method of Walter *et al.* (2005). Crystals for the full-length Adhiron92 scaffold appeared after 38 days in Well H5 of a Morpheus screen (Molecular Dimensions) (0.02 M glycine, 0.02 M sodium l-glutamate, 0.02 M dl-alanine, 0.02 M dl-lysine hydrochloride, 0.02 M dl-serine, 0.1 M MOPS pH 7.50, 0.1 M HEPES sodium salt pH 7.50, 10.0% w/v polyethylene glycol 20 000, 20.0% w/v polyethylene glycol 550 MME). The shorter 81 amino acid Adhiron81 scaffold gave crystals after 52 days in Well D2 of the PACTpremier screen (Molecular Dimensions) (0.1 M Malic acid/MES/Tris Buffer System pH 5.0, 25.0% w/v Polyethylene Glycol 1500).

For the full-length Adhiron92 rod-like crystals ∼25 µm across were cryo-cooled directly into liquid nitrogen without the addition of cryoprotectants. Diffraction data were collected at beamline I24 at Diamond Light Source from multiple positions along a single crystal with the starting angle at each position offset by 30° with respect to the previous position. Data were collected to a resolution of 1.75 Å using an X-ray wavelength of 0.9686 Å with the beamsize set to be 20 × 20 µm^2^ at the sample. For the shorter Adhiron-81 rod-like crystals ∼20 µm across grew in clumps. Diffraction data were collected from a single 20 × 20 × 20 µm^3^ crystal fragment.

All diffraction data were integrated using XDS (Kabsch, 2010) and scaled using AIMLESS (Evans, 2006). Phases were obtained via molecular replacement using PHASER (McCoy *et al.*, 2007). A subsection of a complex of an Adhiron bound to a soluble protein (manuscript in preparation) was used as a search model. Refinement was carried out using REFMAC (Murshudov *et al.*, 2011), PHENIX (Afonine *et al.*, 2012) and COOT (Emsley *et al.*, 2010). Full scaling and refinement statistics are presented in Tables I and II.

**Table I.** Scaling and refinement statistics for Adhiron92

Average unit cell	35.86, 35.86, 61.85; 90, 90, 90		
Space group	*P*4_1_		
	Overall	Inner shell	Outer shell
Low resolution limit	35.86	35.86	1.78
High resolution limit	1.75	9.09	1.75
Rmerge (all I+ and I−)	0.082	0.104	0.897
Rmeas (all I+ and I−)	0.085	0.108	0.960
Rpim (all I+ and I−)	0.023	0.029	0.326
Rmerge in top intensity bin	0.066	–	–
Total number of obs	101 109	737	3060
Total number unique	7889	62	398
Mean ((I)/sd(I))	18.9	38.3	2.6
Completeness	99.3	99.5	91.6
Multiplicity	12.8	11.9	7.7
Average mosaicity	0.14		
Estimated B factor	27.6		
Rwork/Rfree	0.1829/0.2287		
RMS bonds	0.007 Å		
RMS angles	1.072°		
Residues in the favoured region of Ramachandran plot	97.4% (75/77)

**Table II.** Scaling and refinement statistics for Adhiron81

Average unit cell	36.37, 36.37, 59.24; 90, 90, 90		
Space group	*P*4_1_		
	Overall	Inner shell	Outer shell
Low resolution limit	36.37	36.37	2.32
High resolution limit	2.25	9.00	2.25
Rmerge (all I+ and I−)	0.102	0.053	0.722
Rmeas (all I+ and I−)	0.119	0.062	0.839
Rpim (all I+ and I−)	0.060	0.031	0.423
Rmerge in top intensity bin	0.053	–	–
Total number of obs	13 790	209	1295
Total number unique	3709	64	339
Mean((I)/sd(I))	8.2	17.5	1.8
Completeness	99.9	99.5	100.0
Multiplicity	3.7	3.3	3.8
Average mosaicity	0.21		
Estimated Bfactor	33.7		
Rwork/Rfree	0.1839/0.2477		
RMS bonds	0.009 Å		
RMS angles	1.284°		
Residues in the favoured region of Ramachandran plot	93.5% (72/77)		

## Results and discussion

### Consensus phytocystatin

A consensus phytocystatin sequence was derived by identifying the most common amino acid at each position of an alignment of 57 phytocystatin sequences. The length of the initial consensus protein was set at 95 amino acids with the N-terminus positioned four residues before the conserved N-terminal glycine residue, and thus before the first β-strand (β1). The C-terminus was set 15 residues after the conserved PW motif and thus after the last β-strand (β4). These criteria were based on the structures of chicken egg white cystatin (Bode *et al.*, 1988) and human stefin B (Stubbs *et al.*, 1990) from X-ray structures and of OSA-I from the nuclear magnetic resonance structure (Nagata *et al.*, 2000).

### Adhiron scaffold design and phage display

The Adhiron scaffold was derived from the phytocystatin consensus protein by initial N-terminal truncation to a 92 amino acid sequence which we refer to as the full-length Adhiron scaffold, or Adhiron92. We subsequently generated further N-terminal truncated versions of which the shortest is an 81 amino acid sequence referred to as Adhiron81. The coding sequence for the full-length Adhiron, codon optimised to enhance expression in *E. coli* (Fig. 1A), was cloned into the phagemid vector pBSTG1 (Fig. 1B) to allow production of an Adhiron/truncated-pIII fusion protein in ER2738 suppressor cells for phage display.

**Fig. 2.**
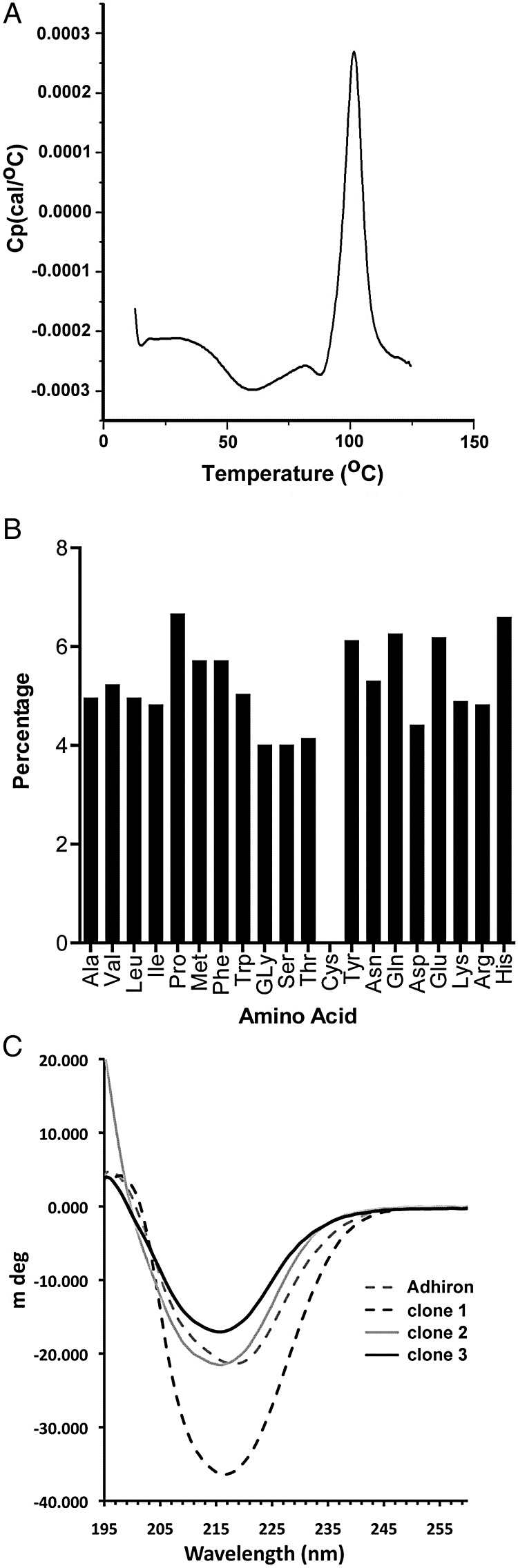
Characterisation of the Adhiron scaffold and library. (**A**) DSC to determine the melting temperature of the Adhiron scaffold (*T*_m_ 101°C). (**B**) Ninety-six random clones were isolated and sequenced both as the phagemid transformed and Adhiron phage library infected *E. coli* ER2738 cells. The percentage of each amino acid within the variable regions is shown. An ideal library would contain 5.26 ± 2.3% of each amino acid; cysteine was not included in the library. (**C**) CD analysis of the Adhiron scaffold and of three Adhiron proteins containing inserts from the library. All show high β structure content.

Expression of the Adhiron-pIII fusion protein was confirmed by Western blot analysis with an anti-pIII antibody. The thermal stability of the Adhiron92 and Adhiron81 scaffolds was tested by DSC and both gave identical profiles with a melting temperature of 101°C (Fig. 2A). The secondary structure was examined by CD, and revealed a high ratio of β sheet to alpha helix and random coil (Fig. 2B). Electrospray mass spectrometry of Adhirons expressed from pET11 showed the predominant species to be lacking only the N-terminal Met, a common modification of cytoplasmically expressed proteins, with a minor component lacking MetAla.

### Adhiron structure

**Fig. 3.**
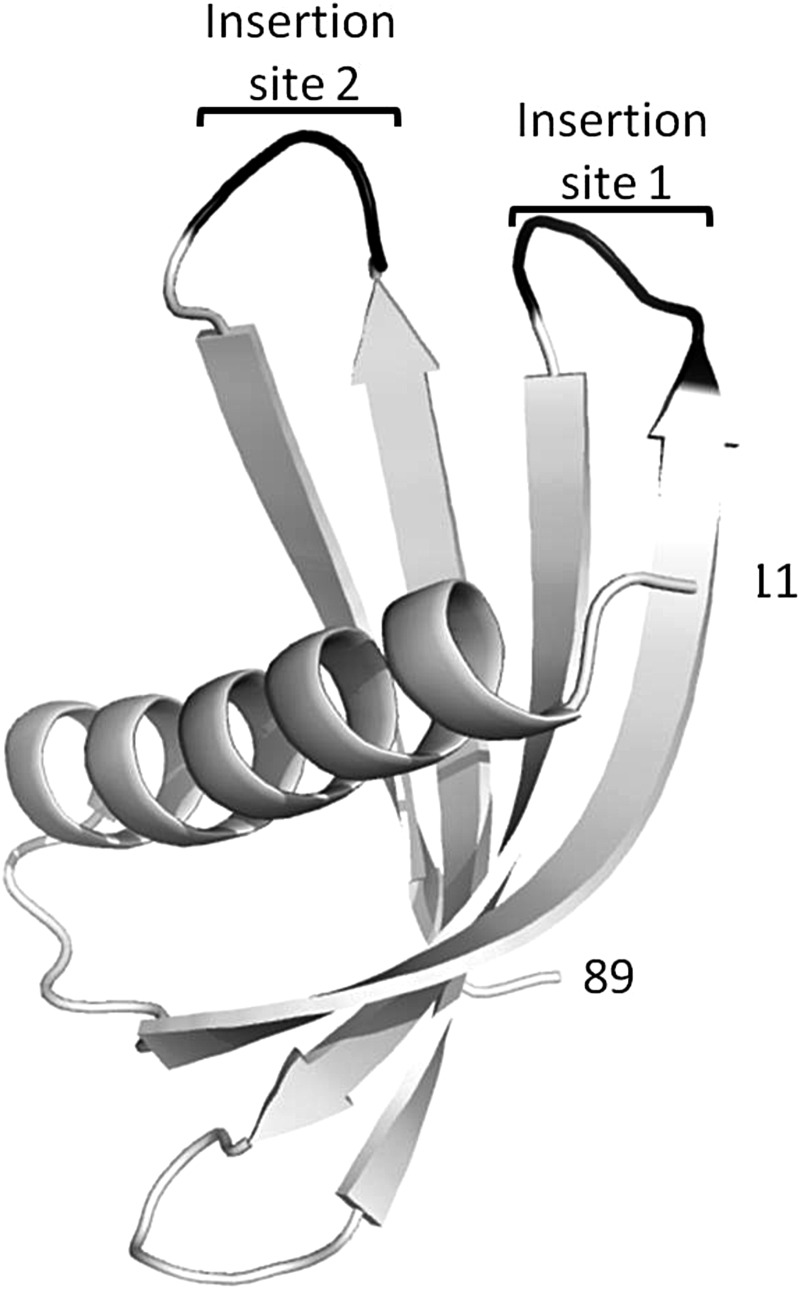
X-ray crystal structure of Adhiron92 scaffold (PDB ID no. 4N6T) at 1.75 Å resolution. The single alpha helix and the four anti-parallel β strands are shown in white with the insertion sites for library production shown in black. Residues 1–10 and 90–92 are not visible in the structure and are presumably disordered. The structure of Adhiron81 at 2.25 Å resolution (PDB ID no. 4N6U) is essentially identical.

We have determined the crystal structure of the full-length Adhiron92 scaffold by X-ray crystallography. The crystals belonged to space group of *P*4_1_ and diffracted to a resolution of 1.75 Å (Table I; PDB ID code 4N6T). The overall structure comprises the characteristic cystatin family fold of a four-strand anti-parallel β-sheet core with a central helix (Fig. 3). Amino acids 1–10 and 90–92 are not visible in the electron density maps presumably as they are disordered. The shorter Adhiron81 also crystallised in space group *P*4_1_ and the structure determined to 2.25 Å resolution (Table II; PDB ID code 4N6U) was essentially identical to that of Adhiron92. The Adhiron structure is compact with limited unstructured loops and this is consistent with the very high melting temperature of this consensus protein.

### Library design

The introduction of peptide encoding sequences, suitable for molecular recognition, was guided by the predicted loop positions within the known structure of the rice phytocystatin OC-1 (PDB code 1EQK). VR 1 was positioned between the first and second β strands with VR 2 between the third and fourth β strands (Fig. 3). Sequences encoding nine random amino acids (excluding cysteine) were introduced at Loops 1 and 2 by replacing four and three amino acid codons, respectively, by using codon-selected semi-trinucleotide cassette synthesis. The phage-display library was estimated to comprise ∼1.3 × 10^10^ independent clones obtained with just 12.6 µg of ligated DNA attributable to high ligation and electroporation efficiencies. To check the amino acid composition, 96 random clones were selected as colonies from the original transformation and revealed no bias in amino acid composition and ca. 94% full-length clones. Following phage recovery and library re-infection of ER2738 cells, a further 96 clones were randomly sequenced. The amino acid residue frequency (Fig. 2B) encoded at the phage level met the expected Poisson statistics of 5.26 ± 2.3% for trimer synthesized oligos using a 19-aa mixture (Krumpe *et al.*, 2007). Clones (86.5%) were full length with only 3.1% clones comprising the Adhiron scaffold with no inserts, and 10.4% clones showing frame shifts. Interestingly, all frame-shift mutants analysed occurred at the transition base between standard nucleotide and trimer coupling, suggesting that this step of DNA synthesis is of crucial importance for semi-trimer oligo synthesis. Fully trimer synthesised oligos might therefore be expected to further improve library quality. However, of the insertions and deletions, between 3 and 8% were unlikely to impact on Adhiron function as the affected variants remain in frame. The high proportion of full-length coding regions at the level of phagemid following phage packaging demonstrates the high quality of the library generated.

Three random Adhiron clones containing inserts were used for protein purification and CD was performed (Fig. 2C). All three showed a high proportion of β structure, as found in parental phytocystatins (Irene D *et al.*, 2012), with one protein displaying a higher content of β structure likely indicating extension of the β strands from the scaffold into the insert regions. This demonstrates that the scaffold can tolerate insertion of peptides in these loop regions and these do not disrupt the secondary structure of the scaffold.

### Library screening

**Fig. 4.**
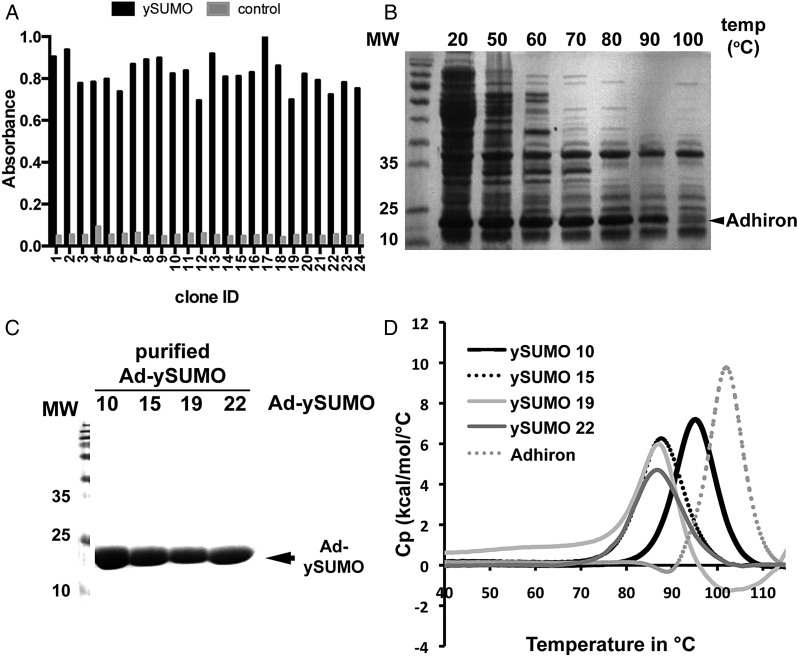
Isolation and purification of yeast SUMO-binding Adhirons. (**A**) Phage ELISA of Adhirons from 24 clones incubated in wells containing ySUMO (black) or control (grey) showing the TMB product absorbance at 560 nm after 3 min. incubation. (**B**) Ad-ySUMOs were expressed in BL21 (DE3) cells and cell lysates were heated to 20, 50, 60, 70, 80, 90 and 100°C for 20 min then 5 µl of cleared lysates was separated by 15% SDS–PAGE and Coomasie stained. (**C**) Ad-ySUMO purification by Ni-NTA beads and analysis of the purified Ad-ySUMOs by 15% SDS–PAGE with Coomassie staining. (**D**) DSC of Ad-ySUMO clones 10, 15, 20 and 22 together with the Adhiron scaffold.

For library evaluation, the ySUMO protein was used as a model target. Over 1000-fold amplification in colony recovery was observed compared with control samples by panning round three. Twenty-four Adhirons were isolated and their ability to bind to ySUMO was confirmed by phage ELISA (Fig. 4A) with little or no binding to the control wells. The clones were sequenced and 22 distinct Adhirons termed Adh-ySUMO 1–22 were identified with the amino acid sequences of the VRs shown in Table III.

**Table III.** Adhiron insert sequences for 24 yeast SUMO-binding Adhirons

Ad-ySUMO	LOOP1	LOOP2
1	WDLTGNVDT	WDDWGERFW
2	IDLTNSFAS	DINQYWHSM
3	INLMMVSPM	GIQQNPSHA
4	IDLTHSLNY	GLTNEIQKM
5	IDLTHSLNY	GLTNEIQKM
6	IDLTEWQDR	PEPIHSHHS
7	WVDMDYYWR	MDEIWAEYA
8	IDLTQTEIV	EPGIIPIVH
9	IDLTDVWID	GLMTQTNSM
10	IIIHENDAD	GIMDGLNKY
11	WILNNTQFI	VLEGPDRWTV
12	WYERSENWD	RDYGFTLVP
13	WDLTTPINI	YEDYQTPMY
14	WFDDEYDWI	DYAATDLYW
15	IDLTQPHDS	YEEDEYWRM
16	IDLTQSFDM	PIDSNFTGT
17	WYLLDVMDD	HDRRYKQAE
18	WIDRGQYWD	IHNGYTIMD
19	WSEADNDWH	LDLETWQHF
20	IDLTGQWLF	PLWQYDAQY
21	IDLTQSFDM	PSHHNYQTM
22	IDLTQSFDM	PIDSNFTGT
23	IDLTQPHDS	PHDELNWNM
24	WEDFQTHWE	DVGQLLSGI

Ad-ySUMO 4 and 5 are identical and 16 and 22 are also identical. Interestingly Ad-ySUMO 15 and 23, as well as 21 and 22, contain the same amino acid sequence in VR 1 but different sequences in VR 2. Analysis of the sequences allowed identification of a commonly occurring SUMO Interacting Motif (SIM) (Kerscher, 2007; Li *et al.*, 2010; Sun and Hunter, 2012) sequence IDLT in Positions 1–4 of VR 1 in 12 of the Ad-ySUMOs, indicating that this is likely to be an important motif in binding to at least one epitope on ySUMO. This SIM motif was not found in VR 2 in any of the clones analysed. Interestingly, the IDLT motif is similar to the human SUMO 1 binding site of the MEF2 E3 ligase PIASx (VDVIDLT) (Song *et al.*, 2004; Song *et al.*, 2005). Either a P or G was identified at Position 1 of VR 2 in nine different Adhirons while a P or G occurs in a position between Residues 2–5 in another 6 Adhirons potentially indicating that some structural feature may be important in binding. For example, clones ySUMO21, 22, 23 and 24 which have the IDLT motif in VR 1 have the motif PX_1–3_(N/Q)(W/F/Y) or G(L,I), which is not found in VR 1. We also identified a common pattern (W/F/Y)(E/D)_2–4_(W/F/Y) represented in both VRs. This molecular pattern matches the criteria for SIMs; a high percentage of hydrophobic amino acids juxtaposed with acidic residues (Song *et al.*, 2005), suggesting that both VRs may act independently, but contribute to the overall binding.

Four Ad-ySUMOs were selected for further characterisation, Clones 15 and 22 as the VR 1 sequence occurred multiple times, Clone 10 as it contained the IDLT motif and Clone 19 as it contained a distinct motif in VR 1.

### Characterisation of the ySUMO Adhirons (Ad-ySUMO)

Due to the high thermal stability of the Adhiron scaffold (Fig. 2A), we predicted that purification may be aided by introducing a heating step to denature and precipitate the majority of *E. coli* proteins without affecting Adhiron integrity. We therefore heated lysates for 20 min at 20, 50, 60, 70, 80, 90 and 100°C, centrifuged to pellet the denatured protein and analysed the supernatants by SDS–PAGE (Fig. 4B). The heating step dramatically decreased the quantity of bacterial protein but did not significantly reduce Adhiron levels. A temperature of 50°C was suitable to remove the majority of bacterial proteins and so was adopted. Figure 4C demonstrates that the purified Ad-ySUMOs show high purity using a batch metal affinity purification method. The estimated level of Adhiron expressed was ∼100 mg/l. DSC confirmed the selected Adhirons maintained heat stability, showing *T*_m_'s of 95.2, 87.7, 87 and 86.7°C for Ad-ySUMO 10, 15, 19 and 22, respectively (Fig. 4D). This demonstrates the ability of the scaffold to effectively constrain the VRs while maintaining high thermal stability of different Adhiron variants. This is consistent with previous observations reported for DARPins that the introduction of diversity results in some loss of stability (Binz *et al.*, 2003; Kohl *et al.*, 2003; Wetzel *et al.*, 2008). However, unlike DARPins we are introducing 18 variable amino acid residues by replacing two short loops of a non-repeat protein scaffold and so the retention of such high thermostability is notable. Fibronectins and the leucine-rich repeat-based Repebodies have reported melting temperatures of around 90°C (Jacobs *et al.*, 2012) and 85°C (Lee *et al.*, 2012), respectively.

**Fig. 5.**
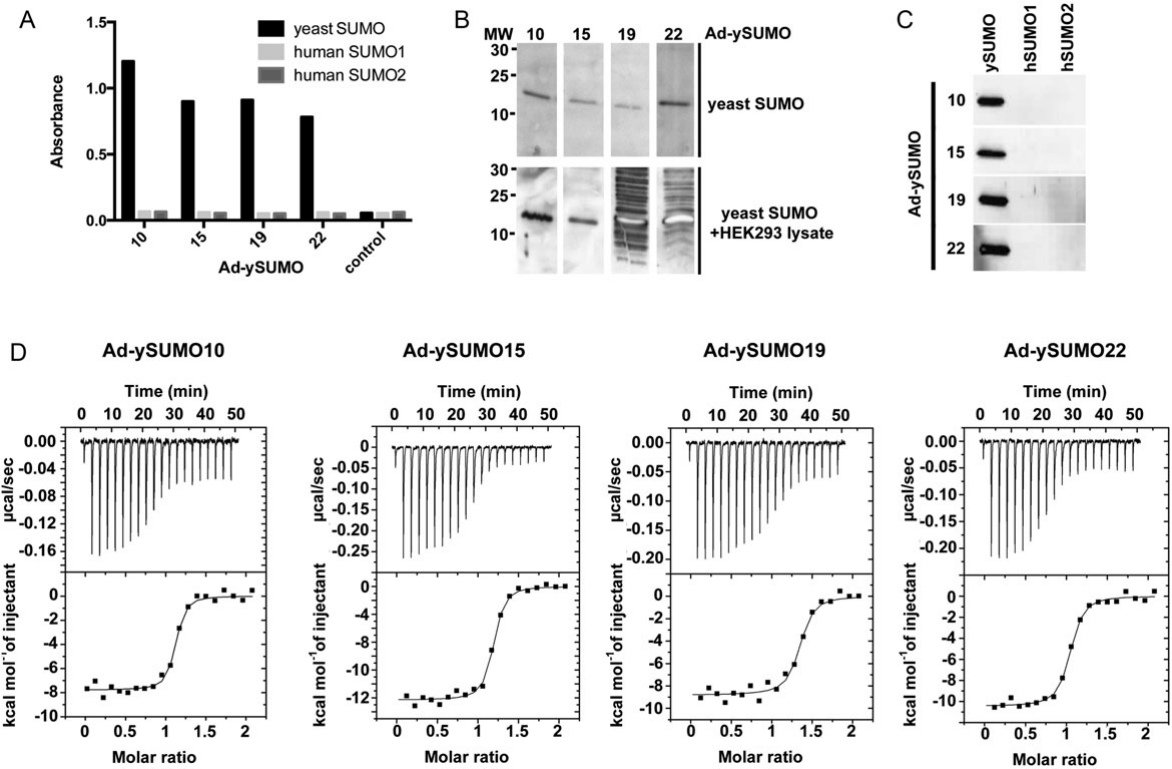
Characterisation of yeast SUMO-binding Adhirons, Ad-ySUMO 10, 15, 20 and 22. (**A**) Biotinylated Ad-ySUMOs were used to detect ySUMO (black). Human SUMO 1 (light grey) and human SUMO 2 (dark grey) by ELISA with TMB product detected at 560 nm. (**B**) Western blots using biotinylated Ad-ySUMO clones against yeast SUMO alone (upper panel) and mixed with 20 µg of HEK293 cell lysate (lower panel). (**C**) Western blot analysis using biotinylated Ad-ySUMO clones against yeast and human SUMOs 1 and 2. (**D**) Isothermal calorimetry of Ad-ySUMOs binding to yeast SUMO with the isotherms and the data fits.

To evaluate the use of the Adhirons as research reagents, the Ad-ySUMOs were biotinylated and used in ELISA (Fig. 5A) and western blot analysis (Fig. 5B and C). The Ad-ySUMOs show selectivity by binding to yeast SUMO but not to human SUMO 1 or 2 (*n* = 3). To determine the specificity of the reagents, yeast SUMO was mixed with HEK293 cell lysates. Interestingly, Ad-ySUMO 10 and 15 show specific binding to yeast SUMO with no binding to other proteins whereas Ad-ySUMO 19 and 22 bind to other proteins in the lysates (*n* = 3), indicating that the inserted sequences are more promiscuous, emphasising the importance of strict negative screens during panning. The affinities of the Adhirons for ySUMO were measured by isothermal titration calorimetry (ITC). Ad-ySUMO 10, 15, 20 and 22 bound with *K*_D_ values of 29.6 ± 0.8, 33.3 ± 1.8, 45.8 ± 8.8 and 47 ± 15.6 nM, respectively (Fig. 5D). Similar affinities were observed using the Blitz™ analysis (ForteBio) when the Adhirons were immobilised on a biosensor surface which validates the use of the Blitz™ for rapid estimation of binding affinity ranges. These *K*_D_ values are in line with those observed for antibodies. By contrast, the *K*_D_ values for human SUMO 1 and 2 were >10^3^-fold higher at 15 and 50 µM, respectively (data not shown). The binding affinities of human SUMO 1 and 2 to a peptide bearing the conserved IDLT SIM motif have been reported to be in the range of 5–6 µM (Song *et al.*, 2005; Li *et al.*, 2010), which is in agreement with our data. The higher affinity for yeast SUMO than the human SUMOs indicates that sequences other than the IDLT in VR 1 and/or 2 play a discriminating role in ySUMO binding. The affinities obtained for the yeast SUMO binders are in the same range as those against other targets reported for DARpins (Schweizer *et al.*, 2007; Steiner *et al.*, 2008) and monobodies (Gilbreth *et al.*, 2011; Grebien *et al.*, 2011), suggesting that the Adhiron scaffold is competitive with other non-antibody binding proteins.

### Further example screens

**Fig. 6.**
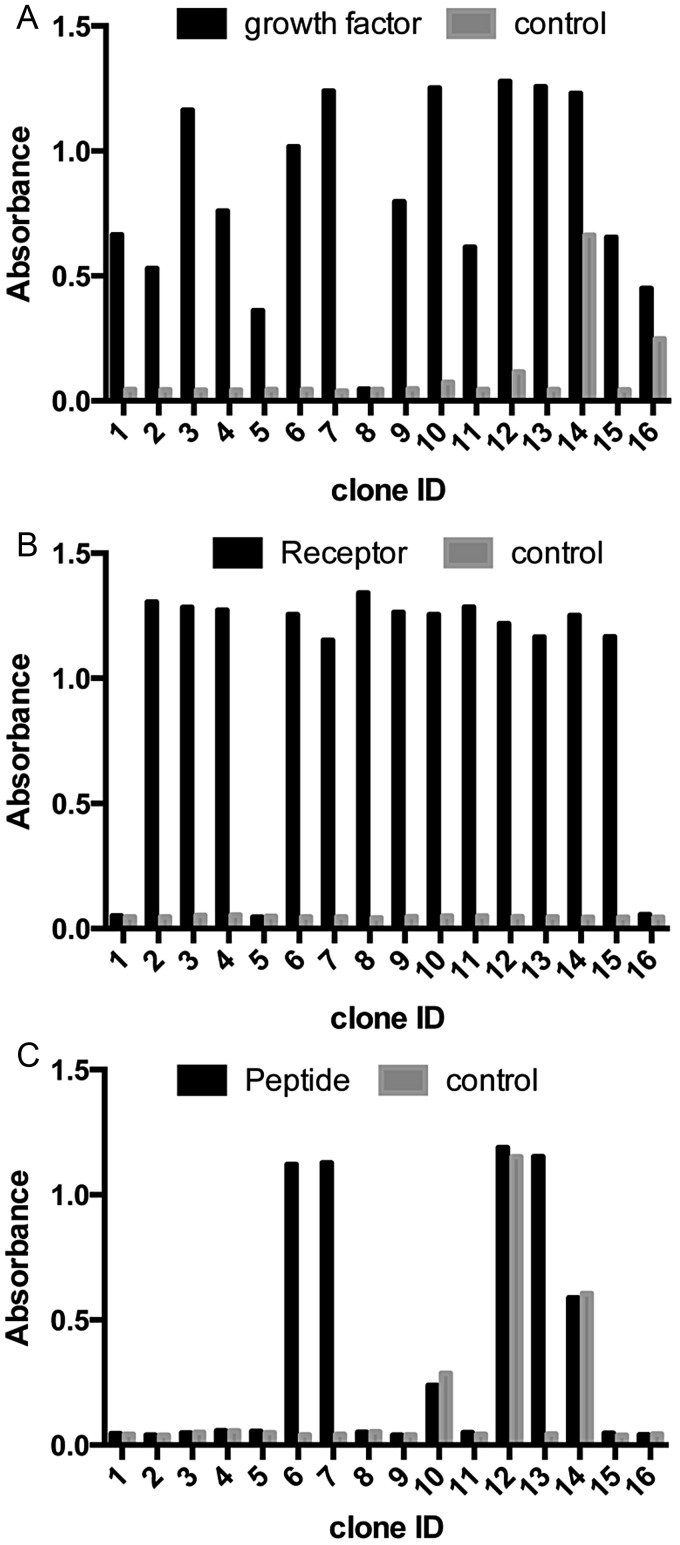
Phage ELISA results for Adhirons identified in screens against (**A**) growth factor protein FGF1, (**B**) cell surface receptor CD31 and (**C**) a peptide. Graphs show absorbance readings of each well after the addition of TMB. Wells containing the target molecule are shown in black and control wells are shown in grey.

To further evaluate the functionality of the Adhiron library, we identified binders to other targets, including the growth factor (FGF1), receptor (CD31), SH2 domain Grb2 and a peptide sequence. All screens were performed over three panning rounds. Phage ELISA was used to examine the ability of recovered Adhirons to bind to the corresponding target (Fig. 6). While the majority of the FGF1, CD31 and Grb2 Adhirons showed specific binding, this was not the case for the peptide screen. In this case, specificity was enhanced by increasing the number of panning rounds. This is not unexpected due to the small size and limited likelihood of appropriate epitope presentation by the peptide compared with larger proteins likely to allow better presentation of single or multiple epitopes. To further confirm that expressed Adhirons bind to their targets, we have used the Blitz™ to analyse three distinct recombinant Adhirons for both CD31 and the peptide target. The Adhirons were expressed and purified as soluble proteins. The *K*_D_ values for CD31 Adhirons ranged from 8.5 × 10^−8^ to 6.8 × 10^−9^ M, while those for the peptide ranged from 3.3 × 10^−8^ to 3.5 × 10^−8^. These data further demonstrate that the phage ELISA identifies high affinity Adhirons.

Analysis of our screen against Grb2 offered the opportunity to directly compare the efficacy of our Adhiron library against well-established ScFv and Fab libraries. A recent study reported high throughput screens of 20 SH2 domains using both hybridoma and phage-display libraries of ScFv's (Colwill *et al.*, 2011). This allows comparison of the results for Grb2 from this study with our screen against Grb2. The hit rates for two ScFv and a Fab library were 4, 43 and 33% (410 clones screened in total) with 5, 2 and 6 unique clones identified from each screen, respectively. In our screen, the hit rate was 92% with over 30 unique binders, although not all were sequenced. Our Adhiron purification success rate was >95% with typical yields of 50–100 mg/l compared with the reported purification success rate of 80% with yields of 0.6–10 mg/l. This is a limited comparative study but demonstrates the complexity of the Adhiron library and the potential for generating specific protein binding reagents.

## Conclusions

We have developed a new artificial binding protein scaffold termed Adhiron based on a designed consensus phytocystatin protein. The properties of this scaffold also match the criteria proposed for artificial therapeutic proteins (Carter, 2011) although it remains to be determined whether Adhirons will be suitable for such applications. The design of the scaffold and library provides a system based on a highly stable scaffold with extended flexible binding regions that are expected to adapt to form appropriate molecular contacts with a wide range of targets allowing interactions with protein pockets, protein surfaces, peptides and small molecules. The system achieves high-level purification of soluble Adhiron (typically 10–100 mg/l) from *E. coli*, by including a heat enrichment step enabling ease of engineering and manufacture. The scaffold displays high thermostability with a melting temperature of 101°C determined by DSC (Fig. 2A).

We have solved the X-ray crystal structure of the Adhiron scaffold reported here, as well as selected Adhirons in complex with target proteins which will be reported elsewhere. The ability to gain structural information is important for improving our understanding of the molecular interactions of Adhirons with their targets that lead to functional consequences, and to provide a basis for drug design. We are currently exploring the extent to which the Adhiron scaffold may offer a platform for selection of reagents for applications including research tools, diagnostic, imaging, therapeutic agents and for drug discovery.

## Supplementary material

Supplementary material is available at *PEDS* online.

## Conflict of interest

Work reported here is included in a patent application filed by the University of Leeds.

## Funding

This work was supported by the Biotechnology and Biological Sciences Research Council [24/G15882] for the development of the consensus cystatin. The scaffold development work was funded by University of Leeds Transformation Fund support for the Biomedical Health Research Centre BioScreening Technology Group. Part of this work concerning characterisation of Adhirons was supported through WELMEC, a Centre of Excellence in Medical Engineering funded by the Wellcome Trust and EPSRC, under grant number WT 088908/Z/09/Z. The OPPF-UK is supported by the MRC and BBSRC. Funding to pay the Open Access publication charges for this article was provided by The Wellcome Trust.

## Supplementary Material

Supplementary Data
