# Supplementary material for: Adhiron: a stable and versatile peptide display scaffold for molecular recognition applications
Source: Protein Eng Des Sel. 2014 Mar 25;27(5):145–55. doi: 10.1093/protein/gzu007 (PMC4000234; doi:10.1093/protein/gzu007)
Supplement: Supplementary Data [file supp_27_5_145__index.html]

Adhiron: a stable and versatile peptide display scaffold for molecular recognition applications — Adhiron: a stable and versatile peptide display scaffold for molecular recognition applications — Supplementary Data 

# Adhiron: a stable and versatile peptide display scaffold for molecular recognition applications

## Supplementary Data

Supplementary Data

**Files in this Data Supplement:**

- Supplementary Data - Doc file
