# Supplementary material for: Adhiron: a stable and versatile peptide display scaffold for molecular recognition applications
Source: Protein Eng Des Sel. 2014 Mar 25;27(5):145–55. doi: 10.1093/protein/gzu007 (PMC4000234; doi:10.1093/protein/gzu007)
Supplement: Supplementary Data [file supp_gzu007_gzu007supp.doc]

**Supplementary Table I. Phytocystatin sequences used to derive the consensus sequence.** Sequences were identified from data bases by homology searching. The table shows a systematic name for each cystatin together with the organism name and common name of the plant and the Genbank accession number.

| **Phytocystatin** | Organism name | **Common name** | **Accession number** |
| --- | --- | --- | --- |
| aar | *Ambrosia artemisiifolia* | Short ragweed | l16624 |
| ace | *Allium cepa* | Onion | aa508918 |
| ath1 | *Arabidopsis thaliana* | Arabadopsis | z17618 |
| ath2 | *Arabidopsis thaliana* | Arabadopsis | z97341 |
| ath3 | *Arabidopsis thaliana* | Arabadopsis | z17675 |
| ath4 | *Arabidopsis thaliana* | Arabadopsis | ataj110 |
| ath6 | *Arabidopsis thaliana* | Arabadopsis | ac002409 |
| ath8 | *Arabidopsis thaliana* | Arabadopsis | z37263 |
| avu | *Artemisia vulgaris* | Mugwort | af143677 |
| bca1 | *Brassica campestris* | Chinese cabbage | l41355 |
| bca2 | *Brassica campestris* | Chinese cabbage | l48182 |
| bca3 | *Brassica campestris* | Chinese cabbage | u51119 |
| cpa | *Carcia papaya* | Papaya | x71124 |
| csa | *Cucumis sativus* | Cucumber | ab014760 |
| csat | *Castanea sativa* | Chestnut | aj224331 |
| cun | *Citrus unshiu* | Satsuma orange | c95263 |
| dca | *Daucus carota* | Carrot | d85623 |
| dcar | *Dianthus caryophyllus* | Carnation | af064734 |
| ghi2 | *Gossypium hirsutum* | Cotton | ai728662 |
| ghi3 | *Gossypium hirsutum* | Cotton | ai726250 |
| gma1 | *Glycine max* | Soybean | d64115 |
| gma2 | *Glycine max* | Soybean | u51583 |
| gma3 | *Glycine max* | Soybean | u51855 |
| gma4 | *Glycine max* | Soybean | u51854 |
| gma5 | *Glycine max* | Soybean | ai495568 |
| gma7 | *Glycine max* | Soybean | ai938438 |
| han1 | *Helianthus annuus* | Sunflower | q10993 |
| iba | *Ipomoea batatas* | Sweet potato | af117334 |
| mcr1 | *Mesembryanthemum crystallinum* | Ice plant | aa856241 |
| mcr2 | *Mesembryanthemum crystallinum* | Ice plant | aa887617 |
| mdo | *Malus domestica* | Apple tree | at000283 |
| osa1 | *Oryza sativa* | Rice | u54702 |
| osa2 | *Oryza sativa* | Rice | x57658 |
| osa5 | *Oryza sativa* | Rice | c25431 |
| pam | *Persea americana* | Avacado | jh0269 |
| pba | *Populus balsamifera* | Poplar | ai167046 |
| pco | *Pyrus comunis* | Pear | u82220 |
| pta | *Pinus taeda* | Pine | ai812403 |
| ptr | *Populus tremula* | Poplar | ai162398 |
| rco1 | *Ricinus communis* | Castor bean | z49697 |
| rco2 | *Ricinus communis* | Castor bean | t23262 |
| sbi | *Sorghum bicolor* | Sorghum | x87168 |
| sla | *Silene latifolia* | White campion | z93053 |
| sly1 | *Lycopersicon esculentum* | Tomato | af083253 |
| sly2 | *Lycopersicon esculentum* | Tomato | x73986 |
| sly5 | *Lycopersicon esculentum* | Tomato | ai1781497 |
| stu1 | *Solanum tuberosum* | Potato | l16450 |
| stu2 | *Solanum tuberosum* | Potato | l16450 |
| stu3 | *Solanum tuberosum* | Potato | l16450 |
| stu4 | *Solanum tuberosum* | Potato | l16450 |
| stu5 | *Solanum tuberosum* | Potato | l16450 |
| stu6 | *Solanum tuberosum* | Potato | l16450 |
| stu7 | *Solanum tuberosum* | Potato | l16450 |
| stu8 | *Solanum tuberosum* | Potato | l16450 |
| stu10 | *Solanum tuberosum* | Potato | x74985 |
| vun | *Vigna unguiculata* | Cowpea | z21954 |
| zma1 | *Zea mays* | Maize | d10622 |
| zma2 | *Zea mays* | Maize | d38130 |
| zma4 | *Zea mays* | Maize | ai001246 |
| zma5 | *Zea mays* | Maize | ai740162 |
